# The changing trends of image-guided biopsy of small renal masses before intervention—an analysis of European multinational prospective EuRECA registry

**DOI:** 10.1007/s00330-022-08556-2

**Published:** 2022-02-05

**Authors:** Vinson Wai-Shun Chan, Francis Xavier Keeley, Brunolf Lagerveld, David J. Breen, Alexander King, Tommy Kjærgaard Nielsen, Marco van Strijen, Julien Garnon, Des Alcorn, Ole Graumann, Eric de Kerviler, Patricia Zondervan, Miles Walkden, Giovanni Lughezzani, Tze Min Wah

**Affiliations:** 1 School of Medicine, Faculty of Medicine and Health, University of Leeds, Leeds, UK; 2 Bristol Urological Institute, North Bristol NHS Trust, Bristol, UK; 3 Department of Urology, OLVG, Amsterdam, the Netherlands; 4 Department of Radiology, Southampton University Hospitals, Southampton, UK; 5 Department of Urology, Aarhus University Hospital, Aarhus, Denmark; 6 Department of Radiology, St. Antonius Hospital, Nieuwegein, the Netherlands; 7 Department of Interventional Radiology, Nouvel Hôpital Civil, 1 place de l’Hôpital, 67000 Strasbourg, France; 8 Department of Interventional Radiology, Gartnavel General Hospital, Glasgow, UK; 9 Department of Radiology, Odense University Hospital, Odense, Denmark; 10 Radiology Department, Saint-Louis Hospital, AP-HP, 1 avenue Claude-Vellefaux, 75475 Paris cedex 10, France; 11 Department of Urology, 26066Amsterdam UMC, University of Amsterdam, Amsterdam, the Netherlands; 12 Department of Imaging, University College London Hospitals NHS Foundation Trust, London, UK; 13 Department of Urology, Vita-Salute San Raffaele University, Milan, Italy; 14 Department of Diagnostic and Interventional Radiology, Institute of Oncology, St. James’s University Hospital, Leeds Teaching Hospitals NHS Trust, Leeds, LS9 7TF UK

**Keywords:** Image-guided biopsy, Cryoablation, Renal cell carcinoma, Kidney neoplasms

## Abstract

**Objectives:**

To evaluate the use of pre-cryoablation biopsy for small renal masses (SRMs) and the effects of increasing uptake on histological results of treated SRMs.

**Methods:**

From 2015 to 2019, patients with sporadic T1N0M0 SRMs undergoing percutaneous, laparoscopic, or open cryoablation from 14 European institutions within the European Registry for Renal Cryoablation (EuRECA) were included for the retrospective analysis. Univariate and multivariate logistic models were used to evaluate the trends, histological results, and the factors influencing use of pre-cryoablation biopsy.

**Results:**

In total, 871 patients (median (IQR) age, 69 (14), 298 women) undergoing cryoablation were evaluated. The use of pre-cryoablation biopsy has significantly increased from 42% (65/156) in 2015 to 72% (88/122) in 2019 (*p* < 0.001). Patients treated for a benign histology are significantly more likely to have presented later in the trend, where pre-cryoablation biopsy is more prevalent (OR: 0.64, 95% CI 0.51–0.81, *p* < 0.001). Patients treated for undiagnosed histology are also significantly less likely to have presented in 2018 compared to 2016 (OR 0.31, 95% CI 0.10–0.97, *p* = 0.044). Patients aged 70+ are less likely to be biopsies pre-cryoablation (*p* < 0.05). R.E.N.A.L. nephrometry score of 10+ and a Charlson Comorbidity Index > 1 are factors associated with lower likelihood to not have received a pre-cryoablation biopsy (*p* < 0.05).

**Conclusion:**

An increased use of pre-cryoablation biopsy was observed and cryoablation patients treated with a benign histology are more likely to have presented in periods where pre-cryoablation biopsy is not as prevalent. Comparative studies are needed to draw definitive conclusions on the effect of pre-cryoablation biopsy on SRM treatments.

**Key Points:**

• *The use of biopsy pre-ablation session has increased significantly from 42% of all patients in 2015 to 74% in 2019*.

• *Patients are less likely to be treated for a benign tumour if they presented later in the trend, where pre-cryoablation biopsy is more prevalent, compared to later in the trend (OR 0.64, 95% CI 0.51–0.81, p < 0.001)*.

• *Patients with comorbidities or a complex tumour (R.E.N.A.L. nephrometry score > 10) are less likely to not undergo biopsy as a separate session to cryoablation*.

**Supplementary Information:**

The online version contains supplementary material available at 10.1007/s00330-022-08556-2.

## Introduction

Renal cancer makes up approximately 2% of all cancers worldwide [1], accounting to almost 40,000 deaths within the European Union in 2018 [2]. The increase in incidental detection of small renal masses (SRMs) over the last decade was said to be attributed to increased routine imaging [3]; the common managements for SRMs include partial nephrectomy, image-guided ablation, and active surveillance, but the optimal diagnostic pathways for SRMs are, however, constantly debated. Currently, the diagnosis of renal tumours is largely based on contrast-enhanced imaging such as computed tomography (CT) or magnetic resonance imaging (MRI) [4]. However, concerns of overtreating benign tumours have arisen due to the high percentage of benign tumours in patients presenting with SRMs. In surgical patients, a large study of 18,060 patients undergoing partial nephrectomy suggested a benign rate as high as 30% [5], while the benign rate can be as high as 26% in mixed treatment patients [6].

A long-term experience by Richard et al found pre-treatment biopsies to be 90% diagnostic, with 26% having benign histology [6]. Similarly, a multicentre study has concluded that routine biopsies reveal significantly lower rates of benign tumours when compared to selected biopsies at the time of surgery [7]. Previous reports of pre-ablation biopsy of small renal tumours showed a benign rate of 18.2%, while a comparator group undergoing biopsy at the time of ablation had a benign rate of 16.8% [8]. These study results has led to the consideration of significant overtreatment and unnecessary surgery for patients with benign tumours as most benign tumours can be safely observed under surveillance due to slow growth rates [9]. In concordance, the latest update of the European Association of Urology (EAU) guidelines strongly suggests the performance of percutaneous renal mass biopsy prior to ablative therapies prior to, but not concomitantly with ablation [10].

A recent meta-analysis by Marconi et al has established the high diagnostic yield and safety profile of image-guided percutaneous biopsy in 2016 [11]; however, the resistance within the urological community to perform renal tumour biopsy remains apparent, due to concerns on the effects of biopsies on altering management plans [12]. Hence, this study, utilising a multicentre European prospectively maintained database, aims to investigate the trends of biopsies pre-cryoablation and their histological results in the past 6 years, and the potential factors influencing the decision to perform image-guided biopsies before active treatment.

## Methods and materials

### Patient selection

Institutional review board approval and patient consent were not required for this registry-based study. The prospectively maintained multicentre European Registry for Renal Cryoablation (EuRECA) [13] was retrospectively enquired to identify patients with primary, sporadic, and localised cT1aN0M0 or cT1bN0M0 SRMs treated by percutaneous, laparoscopic, or open cryoablation at 14 centres around Europe from 2014 to 2020. Patients with cT1a renal masses were defined as a maximum tumour diameter of ≤ 4 cm while cT1b renal masses were defined as > 4 cm and ≤ 7 cm on radiographic imaging according to the American Joint Committee on Cancer (AJCC) staging manual [14]. Patients with multiple renal tumours, recurrences, and inherited renal cell carcinoma (RCC) syndromes were excluded from the analysis [15]. Patients with history of partial nephrectomy, cryoablation, or radiofrequency ablation of the same or contralateral kidney were also excluded from analysis. Due to lack of a full year data, and the COVID-19 pandemic [16–18], patients in 2014 and 2020 were excluded from the analysis. Patients with missing histological results are included in the primary objective, but not the secondary outcome.

### Clinical features and covariates

Patient clinical features including age, sex, race, comorbidities (Charlson Comorbidity Index [CCI] [19]), clinical history, and body mass index were analysed. The baseline estimated glomerular filtration rate (eGFR) derived using the formula from the Modification of Diet in Renal Disease (MDRD) cohort [20] was also collected. Tumour characteristics such as maximum diameter as well as the components of R.E.N.A.L. nephrometry score [21] were collected to identify potential factors influencing the decision of performing pre-cryoablation biopsies.

### Diagnosis and biopsy

Patients were diagnosed of small renal tumours on imaging with either CT, MRI, or ultrasound (US). Patients were then selected to undergo pre-treatment biopsy as a separate session to the treatment session by a multidisciplinary team consisting of urologists, oncologists, and radiologists. Pre-cryoablation biopsies were performed under imaging guidance (US, CT, or MRI). Considering the results from the biopsy (if available), patient’s condition, and preferences, patients were then selected by the multidisciplinary team to undergo cryoablation, other treatments, or active surveillance. Patients with no pre-cryoablation biopsies were biopsied during the cryoablation session using an automated, Tru Cut or suction core device before cryoablation. All biopsies were examined by pathologists at each institution. Biopsies were defined as malignant, benign, and undiagnosed (no classification or normal renal tissue).

### Outcomes and statistical analysis

The primary outcome of the study is to assess the trend of performing image-guided biopsy before cryoablation and the effects of the trend on proportion of patients receiving a definitive pathological diagnosis. The secondary outcome aims to identify potential factors influencing the decision to perform image-guided biopsy before cryoablation. Bar graphs and line charts were utilised to illustrate the change in trend from 2015 to 2019. Univariate logistic regression and chi-squared test were used to assess the effect of the changing trend on performance of pre-cryoablation biopsies and their histological results. Univariate logistic regression and multivariate logistic regression of clinically relevant parameters were performed, taking into account potential confounding biases to assess the factors influencing decision to perform pre-cryoablation biopsy. Both *t*-tests and chi-square tests were utilised to compare baseline characteristics of all patients. All analyses were two-tailed at a significance level of 0.05 and were performed using STATA 16 (Stata Corp) and Microsoft Excel (Microsoft Corp). This study is conducted according to the “Strengthening the Reporting of Observational Studies in Epidemiology” (STROBE) guidelines [22].

## Results

### Baseline characteristics of included patients

**Fig. 1:**
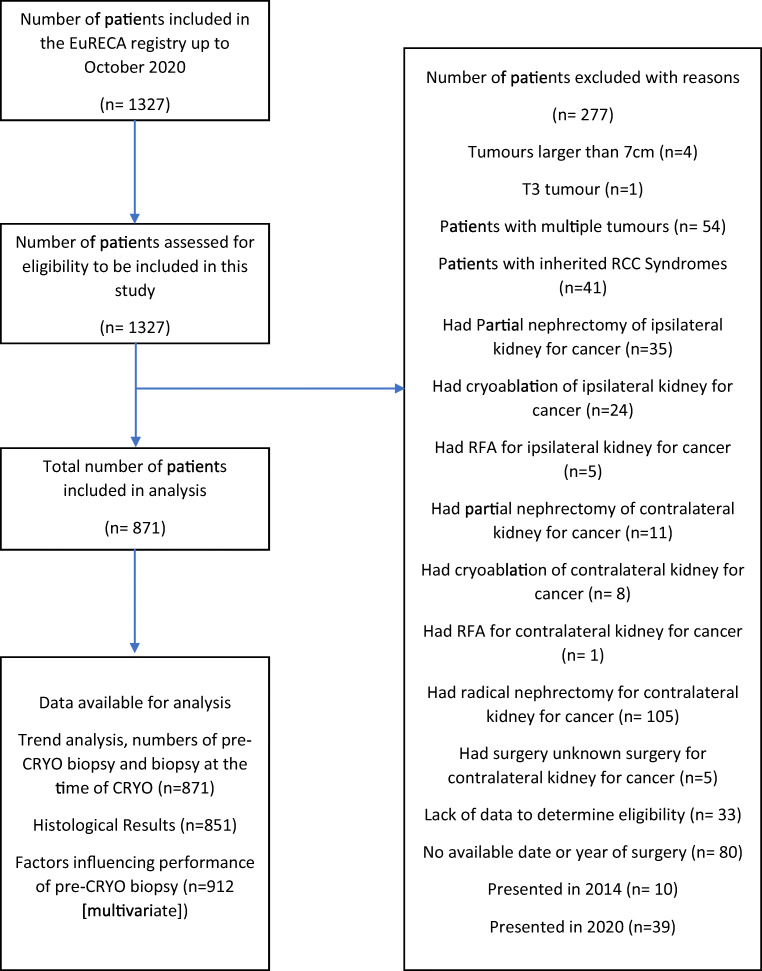
Flow diagram representing the patient selection process

A total of 1327 patients from 14 high-volume, experienced academic centres (Supplementary Table 1) across Europe were included. After applying inclusion and exclusion criteria (Figure 1), 871 patients remained for the retrospective analysis. Out of the 871 patients, 555 (64%; 555/871) received a pre-cryoablation biopsy, whereas 316 (36%; 316/871) did not. The clinical pathologic details of these patients are outlined in Table 1. Amongst the full cohort, 573 are male and 298 are female, dominated by a Caucasian population (96%; 836/871). The median (IQR) age of the cohort is 69 (61–75). A majority of the patients had T1a disease (82.1%; 715/871) and 18% (156/871) had T1b disease. The median (IQR) tumour size of the cohort is 3 cm (2.2–3.6), with a median (IQR) R.E.N.A.L. nephrometry score of 7 (5–8). A total of 4% (37/871) of patients has a solitary functional kidney due to non-cancer causes. The median (IQR) CCI of the cohort is 2 (0–3) with a median baseline eGFR of 85.4 (65.09–107.80) ml/min/1.73 m^2^.

**Table 1:** Clinical and pathological features of included patients

Variable	Presented from 2015 to 2016 (*n* = 348)		Presented from 2017 to 2020 (*n* = 523)		
	Frequency	%	Frequency	%	*p* value
Age					
< 50	29	8.3	32	6.1	0.451
50–59	43	12.4	69	13.2	
60–69	108	31.0	167	31.9	
70–79	125	35.9	194	37.1	
80–89	41	11.8	61	11.7	
> 90	2	0.6	0	0.0	
Sex					
Male	231	66.4	342	65.79	0.764
Female	1117	33.62	181	34.21	
Race					
Caucasian	325	93.4	511	97.7	**0.002**
Asian	9	2.6	8	1.5	
Black	14	4.0	4	0.8	
T-stage (T1a/T1b)					
T1a	284	81.6	431	82.4	0.763
T1b	64	18.48	92	17.6	
Solitary kidney					
Yes	15	4.3	22	4.2	0.941
No	333	95.7	501	95.8	
Received pre-cryoablation biopsy					
Yes	170	48.9	385	73.6	**< 0.001**
No	178	51.2	138	26.4	
Histology					
Malignant	273	82.2	438	91.1	**< 0.001**
Benign	36	10.8	20	4.2	
Undiagnosed	23	6.9	23	4.8	
Missing	18		51		
Variable	Median	IQR	Median	IQR	*p* value
Tumour size (cm)	3.0	2.3–3.6	3.0	2.2–3.6	0.563
R.E.N.A.L. nephrometry score	7	5–8	7	5–8	0.784
Charlson Comorbidity Index	2	0–3	2	1–3	**0.027**
Baseline eGFR	95.2	68.3–150.0	82.1	64.2–97.7	**< 0.001**

*CRYO* cryoablation, *eGFR* estimated glomerular filtration rate

**Bolded** *p* value suggests significance to the level of 0.05

Bolded *p* value suggests significance to the level of 0.05

### Trends of pre-cryoablation biopsy

**Fig. 2:**
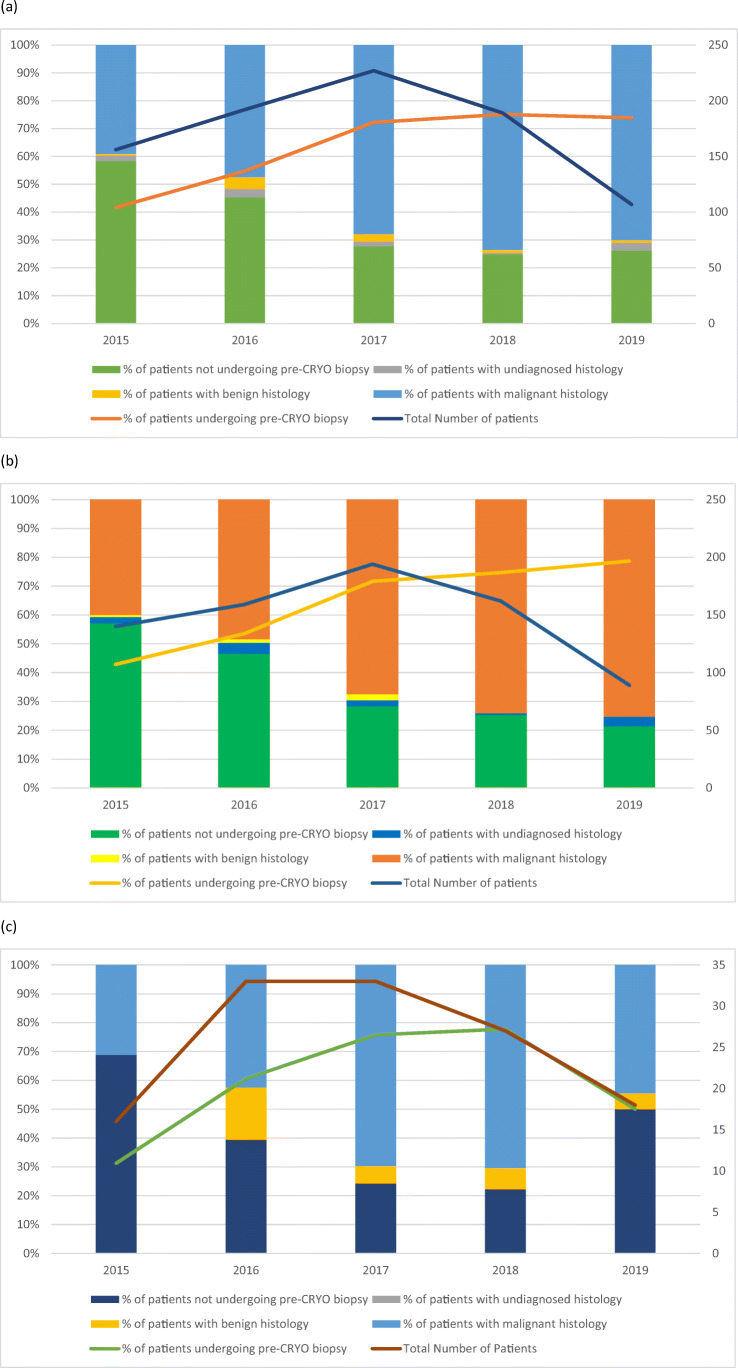
Trends of performance of pre-cryoablation biopsy and the results of histological results from 2014 to 2020 in (**a**) T1 disease, (**b**) T1a disease, and (**c**) T1b disease

A trend of significantly increasing use of pre-cryoablation biopsy was observed between the period of 2015 and 2019 amongst all T1 diseases (Figure 2a; Table 2). The utilisation of pre-cryoablation biopsy has risen from 42% (65/156) in 2015 to 74% (79/107) in 2019 (*p* < 0.001), with a similar trend observed in T1a disease alone (Figure 2b). In T1b disease, the use of pre-cryoablation biopsy rose steadily from 2015 to 2018 and declined in 2019. While the trend does not follow that of T1 and T1a, general increase of usage of pre-cryoablation biopsies for T1b tumours has been observed (Figure 2c). Table 2 shows the breakdown of number of biopsies by year and the results of univariate logistic regression to identify the changing trends in the use of pre-cryoablation biopsy; notably, year by year, patients treated after 2015 are significantly more likely to have received pre-cryoablation biopsy when compared with those who received treatment in 2015.

**Table 2:** Performance of pre-cryoablation biopsy by year and results of univariate logistic regression

Year	Number of pre-CRYO biopsies performed	%	Number of patients with no pre-CRYO biopsy performed	%	Total number of patients	OR (95% CI)	*p* value (logistic regression)	*p* value (chi-squared)
2015	65	41.7%	91	58.3%	156	Ref.		**< 0.001**
2016	105	54.7%	87	45.3%	192	1.69 (1.10–2.59)	**0.016**	
2017	164	72.2%	63	27.8%	227	3.64 (2.37–5.61)	**< 0.001**	
2018	142	75.1%	47	24.9%	189	4.23 (2.67–6.69)	**< 0.001**	
2019	79	73.8%	28	26.2%	107	3.95 (2.31–6.75)	**< 0.001**	
Total	555		316		971	1.51 (1.35–1.70)*	**< 0.001**	

*OR* odds ratio, *CI* confidence interval, *Ref* reference, *N/A* not applicable, *CRYO* cryoablation

*Analysed as with year of surgery as continuous variable across whole study period (2015–2019)

**Bolded** *p* value suggests significance to the level of 0.05

### Histological results of biopsies in relation to time and utilisation of pre-cryoablation biopsy: pre-cryoablation vs at the time of cryoablation

**Fig. 3:**
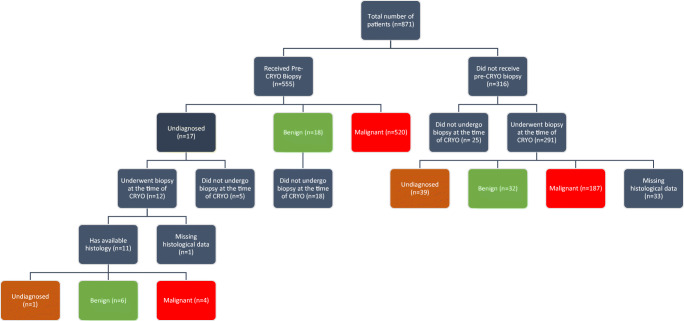
Outline of the diagnostic process and histological results

The diagnostic process and the histological results of the 871 included patients are outlined in Figure 3 and Supplementary Table 2. Amongst the 871 patients, 33 had missing histological data and were excluded from the analysis. Twenty-five patients did not receive a biopsy at the time of cryoablation and was therefore also excluded from the analysis. Amongst the 813 remaining patients, those who presented later in the trend had significantly higher rate of malignancy as compared to those who presented earlier in the trend (OR 1.33, 95% CI 1.12–1.58, *p* = 0.001). Similarly, patients who presented later in the trend had significantly lower rate of benign histology as compared to those who presented earlier in the trend (OR 0.64, 95% CI 0.51–0.81, *p* < 0.001). When comparing the periods of increasing adoption, patients are less likely to receive cryoablation for undiagnosed biopsies (OR 0.31, 95% CI 0.10–0.97, *p* = 0.044) in 2018, using 2016 as a baseline. However, when spanning the full study period from 2015 to 2019, the difference is insignificant (OR 0.93, 95% CI 0.74–1.18, *p* = 0.554), given the anomalously low undiagnosed rate in 2015. The full categorical logistic regression and breakdown of histological outcome per year are shown in Table 3.

**Table 3:** Results of categorical and continuous logistic regression and breakdown of histological result by year of surgery

Year	Undiagnosed	% of all patients in year	OR (95% CI)	*p* value (logistic regression)	*p* value (chi-squared)	Benign	% of all patients in year	OR (95% CI)	*p* value (logistic regression)	*p* value (chi-squared)	Malignant	% of all patients in year	OR (95% CI)	*p* value (logistic regression)	*p* value (chi-squared)	Total number of patients with available histology data
2015	10	6.8	Ref.			21	14.2	Ref.			117	79.1	Ref.			148
2016	13	7.1	1.05 (0.45–2.47)	0.912		15.0	8.2	0.54 (0.27–1.08)	0.082		156	84.8	1.48 (0.84–2.6)	0.176		184
2017	10	4.8	0.7 (0.28–1.73)	0.44		10.0	4.8	0.31 (0.14–0.67)	**0.003**		187	90.3	2.48 (1.35–4.55)	**0.003**		207
2018	4	2.3	0.32 (0.1–1.06)	0.062		6	3.5	0.22 (0.08–0.55)	**0.001**		164	94.3	4.35 (2.05–9.21)	**< 0.001**		174
2019	9	9.0	1.36 (0.53–3.49)	0.516		4	4	0.25 (0.08–0.76)	**0.014**		87	87	1.77 (0.88–3.59)	0.111		100
Total	48	5.6	0.93 (0.74–1.18)*	0.554	0.133	60	7.05	0.64 (0.51–0.81)*	**< 0.001**	**0.001**	743	87.3	1.33 (1.12–1.58)*	**0.001**	**0.001**	851

*OR* odds ratio, *CI* confidence interval, *Ref* reference, *N/A* not applicable, *CRYO* cryoablation

*Analysed with year of surgery as a continuous variable across full study period (2015–2019)

**Bolded** *p* value suggests significance to the level of 0.05

Overall, 6.9% (66/813) and 5.7% (46/813) patients had a benign or undiagnostic histology results, respectively, suggesting a total combined undiagnosed or benign rate of 12.6% (102/831). This rate has decreased significantly from 20.9% (31/148) in 2015 to 5.7% (10/174) in 2018 (*p* < 0.001).

### Factors influencing performance of pre-cryoablation biopsy

Univariate and multivariate logistic regressions were performed to identify potential factors influencing performance of pre-cryoablation ablation (Table 4). Amongst multivariate logistic regression of clinically relevant parameters, it was found that patients aged 70–79 (OR 2.17, 95% CI 1.13–4.18, *p* = 0.020) and 80–89 (OR 2.39, 95% CI 1.14–5.02, *p* = 0.021) are more likely to have not received pre-cryoablation biopsy. In reverse, those with a CCI greater than 1 (OR 0.40, 95% CI 0.28–0.57, *p* < 0.001) or with a R.E.N.A.L. nephrometry score of 10–12 (OR 0.45, 95% CI 0.25–0.84, *p* = 0.011) are less likely to have not received a pre-cryoablation biopsy. When exploring R.E.N.A.L. nephrometry by its components, patients with posterior tumours (OR 0.48, 95% CI 0.34–0.68, *p* < 0.001) or tumours that are less than 50% exophytic (OR 0.67, 95% CI 0.50–0.91, *p* = 0.010) or entirely endophytic (OR 0.55, 95% CI 0.37–0.84, *p* = 0.005) or are not touching renal artery or vein (OR 0.30, 95% CI 0.19–0.50, *p* < 0.001) are less likely to be not biopsied pre-cryoablation. Tumours that cross the polar line more than 50%, crosses the axial line, and between the polar lines are also significantly less likely to be not biopsied before treatment (OR 0.64, 95% CI 0.45–0.89, *p* = 0.008). Patients with lesions that were ≤ 4 mm from the collecting system or sinus are significantly more likely to not be biopsied (1.85, 95% CI 1.34–2.54, *p* < 0.001). Logistic regression of R.E.N.A.L. score and patients’ likelihood to receive pre-cryoablation biopsy are outlined in Table 5. Other factors such as tumour diameter and baseline eGFR were not found to be associated with the odds of patients receiving pre-cryoablation biopsy.

**Table 4:** Univariate and multivariate logistic regression of factors influencing decision to not perform pre-CRYO biopsy

Clinical and pathological characteristic	Univariate OR	Lower 95% CI	Upper 95% CI	*p* value (logistic regression)	*p* value (chi-squared)	Multivariate OR	Lower 95% CI	Upper 95% CI	*p* value (logistic regression)
Age									
< 50	Ref.				0.125	Ref.			
50–59	1.28	0.64	2.57	0.489		1.28	0.62	2.66	0.503
60–69	1.48	0.80	2.77	0.213		1.76	0.91	3.40	0.091
70–79	1.86	1.01	3.43	**0.047**		2.17	1.13	4.18	**0.020**
80–89	2.05	1.03	4.10	**0.042**		2.39	1.14	5.02	**0.021**
90+	Not estimable^a^					Not estimable^a^			
Sex									
Male	Ref.				0.291	Ref.			
Female	0.85	0.64	1.14	0.291		0.78	0.57	1.07	0.122
Race									
Caucasian	Ref.				0.522	Ref.			
Asian	0.53	0.17	1.65	0.275		0.64	0.20	2.02	0.443
Black	0.87	0.32	2.33	0.776		1.02	0.36	2.88	0.976
Charlson Comorbidity Index									
0	Ref.				**< 0.001**	Ref.			
1	0.81	0.52	1.28	0.375		0.71	0.44	1.15	0.166
1+	0.45	0.32	0.61	**< 0.001**		0.40	0.28	0.57	**< 0.001**
Obesity									
No	Ref.				0.062	Ref.			
Yes	1.34	0.98	1.82	0.063		1.35	0.97	1.88	0.072
T-stage									
T1a	Ref.				0.418	N/A^b^			
T1b	1.16	0.81	1.65	0.419					
R.E.N.A.L. score									
4–6	Ref				**0.014**	Ref.			
7–9	1.20	0.90	1.60	0.211		1.13	0.83	1.54	0.436
10–12	0.53	0.29	0.94	**0.029**		0.45	0.25	0.84	**0.011**
Tumour diameter									
< 2 cm	Ref.				**0.025**	Ref.			
2.1–4 cm	1.62	1.12	2.35	**0.011**		1.34	0.90	2.01	0.150
4.1–7 cm	1.72	1.08	2.74	**0.021**		1.52	0.91	2.53	0.111
Solitary functional kidney									
No	Ref.				0.619	Ref.			
Yes	1.19	0.59	2.41	0.619		1.34	0.61	2.94	0.465
Baseline eGFR									
≥ 90	Ref.				0.924	Ref.			
60–89	0.90	0.66	1.23	0.492		0.82	0.58	1.14	0.230
45–59	1.10	0.71	1.72	0.659		1.08	0.67	1.74	0.747
30–44	0.78	0.39	1.56	0.483		1.04	0.50	2.19	0.911
15–29	0.90	0.37	2.17	0.811		1.22	0.48	3.10	0.678
< 15	0.84	0.15	4.65	0.843		0.85	0.15	4.94	0.857

*OR* odds ratio, *CI* confidence interval, *Ref* reference, *eGFR* estimated glomerular filtration rate

^a^Small number in this group does not allow adequate estimation of OR

^b^Excluded from multivariate analysis because of collinearity with tumour diameter

^c^Excluded from multivariate analysis because of collinearity with solitary kidney

**Bolded** *p* value suggests significance to the level of 0.05

**Table 5:** Univariate logistic regression of R.E.N.A.L. nephrometry score components influencing decision to not perform pre-cryoablation biopsy

R.E.N.A.L. component	OR	95% CI	*p* value (logistic regression)	*p* value (chi-squared)
Exophytic/endophytic				
≥ 50% exophytic	Ref.			**0.005**
< 50% exophytic	0.67	0.50–0.91	**0.010**	
Entirely endophytic	0.55	0.37– 0.84	**0.005**	
Nearness to collecting system or sinus (mm)				
≥ 7	Ref.			**< 0.001**
> 4 and < 7	1.06	0.71–1.57	0.776	
≤ 4	1.85	1.34–2.54	**< 0.001**	
Anterior/posterior				
Anterior	Ref.			**< 0.001**
Posterior	0.48	0.34–0.68	**< 0.001**	
Neither	0.73	0.50–1.04	0.078	
Location relative to polar lines				
Entirely above or below	Ref.			**0.022**
Lesion crosses the polar line for < 50%	0.94	0.67–1.31	0.709	
Lesion crosses the polar line for > 50%; lesion crosses the axial line; lesion is between polar lines	0.64	0.45–0.89	**0.008**	
Hilar tumour				
Yes, if tumour touches renal artery or vein	Ref.			**< 0.001**
No	0.30	0.19–0.50	**< 0.001**	

*OR* odds ratio, *CI* confidence interval, *Ref* reference

**Bolded** *p* value suggests significance to the level of 0.05

## Discussion

The EuRECA registry includes high-volume centres across Europe with considerable experience in renal biopsy. This study identified an important paradigm shift of the use of pre-cryoablation biopsy in the past half-decade as utilisation of pre-cryoablation biopsy has risen from 42% (65/156) in 2015 to 74% (79/107) in 2019 (*p* < 0.001), in concordance with the EAU’s advocacy in performing pre-treatment biopsy in SRMs [10]. Other than more informative guidelines and patient-centred care, multiple studies suggesting the safety, diagnostic accuracy, as well as the potential benefits of performing pre-treatment biopsy [23–26] have led to an increase uptake of pre-treatment biopsy. Notably, Maturen et al concluded the definitive role of pre-cryoablation biopsy to significantly alter treatment decisions, especially in benign diseases where patients would have otherwise received a nephrectomy [25] and where low-grade tumours can be safely monitored by active surveillance. In the EuRECA registry, we found patients presented late in the trend, who are more likely to have received pre-cryoablation biopsy, to have a significantly lower benign rate compared to patients who did not (OR 0.64, 95% CI 0.51–0.81, *p* = 0.001), suggesting a potentially significant role of pre-cryoablation biopsy to prevent treatment of patients with benign tumours, understanding that some patients may receive treatment for benign tumours due to their increasing size. In comparison to published data, cohorts not undergoing pre-treatment biopsy have a benign surgical histology of up to 30% [5, 6] compared to 12.4% [32/258] in this cryoablation cohort. While pre-treatment biopsy may be regarded as an extra procedure, the risk of seeding haemorrhage and seeding is minimal. A systematic review reported only one case of transitional cell carcinoma seeding in 5228 biopsies performed, with a bleeding rate of 0.7% [11]. Furthermore, the benefits of renal tumour biopsy do not limit to informing a better treatment plan and to reducing overtreatment; pre-treatment biopsies can also increase the rates of patients receiving definitive histological confirmation after treatment. The EuRECA registry has shown a 15% (39/258) rate of undiagnosed histology at first attempt of biopsy during cryoablation, suggesting these 15% of patients will not receive a histological confirmation. As opposed, if patients do have an undiagnosed pre-cryoablation biopsy, as shown in our data, only 1/11 continued to have undiagnosed histology at the time of cryoablation, suggesting the advantage of the extra pre-cryoablation biopsy step in patient’s treatment pathway. In light of these findings, this study hopes to serve as evidence for the EAU guidelines to recommend pre-ablation biopsy as an optimal step in the patient’s RCC management.

A major strength of this study is the high volume and experience of the included centres. The overall undiagnosed or benign rate is 12.5% (102/813) in the EuRECA registry, which is significantly lower than previous reports [5, 6], although patients with benign tumours may have been excluded from cryoablation treatment in the EuRECA registry. Furthermore, the undiagnosed or benign detection rate has reduced over the years, suggesting a major improvement in the way patients were selected for biopsies and eventually for cryoablation. Our analysis suggests age to be a major factor in influencing the decision of performing a pre-cryoablation biopsy, especially amongst those in their 70s or 80s. Understandably, these patients have lower RCC-specific mortality rates as a result of aging and other potential comorbidities, along with the potential risks to undergo pre-cryoablation biopsy; active surveillance should be advocated for such cases [27–30] as per EAU guidelines [10], as performing a biopsy will not significantly affect the management plan. On the contrary, our study has found patients with a CCI > 1 are less likely not to have received a pre-cryoablation biopsy, while a pre-cryoablation biopsy may put comorbid patients through an extra procedure and cumulative risks; a benign histology may reduce the risk of patients having to receive a potential risk of general anaesthesia during cryoablation session, striking a balance on to treat or not to treat. Patients with a R.E.N.A.L. nephrometry score of over 10 are also less likely to not be biopsied due to the risk of complications associated with high nephrometry score during both nephrectomy and ablation [31, 32].

To our knowledge, this study is the first multicentre study in Europe to investigate the trends of pre-cryoablation biopsy and the factors influencing the performance of pre-cryoablation biopsy. While the results may have provided some insightful and positive findings, it does not come without limitations. Firstly, the EuRECA database only captures data on patients who eventually undergo image-guided cryoablation; hence, this study is unable determine the effect of pre-operative biopsies on treatment decisions. Secondly, this study is limited to patients undergoing cryoablation, and the results may not be generalisable to patients undergoing other forms of treatment, i.e., radiofrequency ablation or partial nephrectomy. However, we believe the principle remains the same in other treatment modalities where pre-treatment biopsy should be obtained pre-procedure. Finally, owing to the multinational design of the study, there may be heterogeneity in decision-making for patients selected for pre-cryoablation biopsies; hence, the results may not be representative of a wider population or at a specific centre. Nonetheless, we hope this study may increase awareness of the accuracy, safety, efficacy, and advocates for the importance of performing pre-treatment biopsy to reduce the over treatment of benign renal masses.

This multicentre analysis has confirmed the changing trend to adopt image-guided biopsy before treatment over the last decade. The likelihood of obtaining confirmatory histological diagnosis and benign histology decreases significantly when biopsy is performed before treatment as a separate session, and the patient should be consented accordingly during consultation to minimise overtreatment of benign tumours.

## Supplementary information

ESM 1 (DOCX 19 kb)

## Abbreviations

CCI: Charlson Comorbidity Index
CT: Computed tomography
EAU: European Association of Urology
eGFR: Estimated glomerular filtration rate
EuRECA: European Registry for Renal Cryoablation
IQR: Interquartile range
MRI: Magnetic resonance imaging
RCC: Renal cell carcinoma
SRM: Small renal mass
US: Ultrasound

## Declarations

### Guarantor

The scientific guarantor of this publication is Mr. Vinson Wai-Shun Chan and Prof. Tze Min Wah.

### Statistics and biometry

No complex statistical methods were necessary for this paper.

### Methodology

• retrospective

• observational

• multicentre study
