# Supplementary material for: The changing trends of image-guided biopsy of small renal masses before intervention—an analysis of European multinational prospective EuRECA registry
Source: Eur Radiol. 2022 Feb 5;32(7):4667–78. doi: 10.1007/s00330-022-08556-2 (PMC8817647; doi:10.1007/s00330-022-08556-2)
Supplement: Supplementary file 1 — (DOCX 19 kb) [file 330_2022_8556_MOESM1_ESM.docx]

| Centre Name | Country | Number of patients included | Percentage |
| --- | --- | --- | --- |
| OLVG location West | Netherlands | 16 | 1.84 |
| OLVG location East | Netherlands | 90 | 10.33 |
| Southmead Hospital | England | 21 | 2.41 |
| St Antonius Ziekenhuis | Netherlands | 35 | 4.02 |
| Aarhus Universitetshospital | Denmark | 162 | 18.60 |
| University Hospital of Southampton | England | 179 | 20.55 |
| Nouvel Hopital Civil, CHU de Strasbourg | France | 83 | 9.53 |
| University College Hospital London | England | 37 | 4.25 |
| Instituto Clinico Humanitas Rozzano | Italy | 7 | 0.80 |
| Odense University Hospital | Denmark | 23 | 2.64 |
| St James University Hospital | England | 159 | 18.25 |
| Gartnavel General Hospital | Scotland | 48 | 5.51 |
| Saint Louis Hospital | France | 7 | 0.80 |
| Amsterdam UMC | Netherlands | 4 | 0.46 |

Supplementary Table 1 – Name, country and number of patients of each included centres

| Histology | Histological Type |
| --- | --- |
| Patients undergoing Pre-CRYO Biopsy (n=555) | |
| Undiagnosed (n=17) |  |
|  | Normal Renal Tissue (n=10) |
|  | No classification (n=7) |
| Benign (n=18) |  |
|  | Oncocytoma (n=5) |
|  | Angiomyolipoma (n=13) |
| Malignant (n=520) |  |
|  | Clear Cell (n=345) |
|  | Chromophobe (n=33) |
|  | Papillary (n=105) |
|  | Spindle Cell (n=3) |
|  | RCC unspecified (n=30) |
|  | Other (n=4) |
|  | |
| Patients undergoing biopsy at the time of CRYO (n= 291) | |
| Undiagnosed (n=39) |  |
|  | Normal Renal Tissue (n=29) |
|  | Fibrous Tissue or Scar (n=9) |
|  | Missing data (n=1) |
| Benign (n=32) |  |
|  | Oncocytoma (n=32) |
| Malignant (n=187) |  |
|  | Clear Cell (n=152) |
|  | Chromophobe (n=14) |
|  | Papillary (n=16) |
|  | RCC unspecified (n=3) |
|  | Other (n=2) |
| Missing histological data (n=33) |  |
|  | |
| Patients undergoing biopsy at the time of CRYO after an undiagnosed pre-CRYO biopsy (n=12) | |
| Undiagnosed (n=1) |  |
|  | Normal Renal Tissue (n=1) |
| Benign (n=6) |  |
|  | Oncocytoma (n=6) |
| Malignant (n=4) |  |
|  | Clear Cell (n=4) |
| Missing histological data (n=1) |  |

Supplementary Table 2 – Detailed histological results of all patients
